# High peritumoral network connectedness in glioblastoma reveals a distinct epigenetic signature and is associated with decreased overall survival

**DOI:** 10.1093/neuonc/noaf101

**Published:** 2025-04-15

**Authors:** Kerstin Jütten, Jonas Ort, Julius M Kernbach, Anke Meyer-Baese, Uwe Meyer-Baese, Hussam Aldin Hamou, Hans Clusmann, Martin Wiesmann, Juliane Bremer, Henner Koch, Aniella Bak, Franz Ricklefs, Richard Drexler, Dieter-Henrik Heiland, Daniel Delev

**Affiliations:** Department of Neurosurgery, RWTH Aachen University, Aachen, Germany; Department of Neurosurgery, RWTH Aachen University, Aachen, Germany; Department of Neurosurgery, RWTH Aachen University, Aachen, Germany; Department of Neuroradiology, University Hospital Heidelberg, Heidelberg, Germany; Department of Electrical and Computer Engineering, FAMU-FSU College of Engineering, Tallahassee, FL, USA; Department of Electrical and Computer Engineering, FAMU-FSU College of Engineering, Tallahassee, FL, USA; Department of Neurosurgery, RWTH Aachen University, Aachen, Germany; Department of Neurosurgery, RWTH Aachen University, Aachen, Germany; Center for Integrated Oncology Aachen Bonn Cologne Duesseldorf (CIO ABCD), Germany; Department of Diagnostic and Interventional Neuroradiology, RWTH Aachen University, Aachen, Germany; Institute of Neuropathology, RWTH Aachen University, Aachen, Germany; Department of Neurology, Section of Epileptology, RWTH Aachen University, Aachen, Germany; Department of Neurology, Section of Epileptology, RWTH Aachen University, Aachen, Germany; Department of Neurosurgery, University Medical Center Hamburg-Eppendorf, Hamburg, Germany; Department of Neurosurgery, University Medical Center Hamburg-Eppendorf, Hamburg, Germany; Department of Neurology and Neurology Sciences, Stanford University, Stanford, CA, USA; Department of Translational Neurosurgery, Friedrich-Alexander University Erlangen-Nürnberg, Erlangen, Germany; Department of Neurosurgery, University Hospital Erlangen, Friedrich-Alexander University Erlangen Nürnberg, Erlangen, Germany; Department of Neurological Surgery, Northwestern University Feinberg School of Medicine, Chicago, IL, USA; Department of Neurosurgery, RWTH Aachen University, Aachen, Germany; Department of Neurosurgery, University Hospital Erlangen, Friedrich-Alexander University Erlangen Nürnberg, Erlangen, Germany

**Keywords:** degree centrality, glioblastoma, network theory, overall survival, resting-state functional connectivity

## Abstract

**Background:**

Glioblastomas are functionally integrated into their peritumoral neural environment, and the dynamic functional interaction can be analyzed using network theory, providing insights into the tumor-brain interface. We investigated the peritumoral network connectedness of glioblastomas, revealing its association with distinct epigenetic signatures, its influence on survival, and its susceptibility to modification through surgical treatment.

**Methods:**

Resting-state fMRI was performed on 48 glioblastoma patients. Tumor lesions were segmented, and networks were constructed at 10 mm and 40 mm distances from the tumor margin. These networks were mirrored to the healthy hemisphere to compare lesional and contralesional networks. The difference between lesional and contralesional mean degree centrality was calculated to assess the peritumoral network connectedness. Its correlation with epigenetic signatures and effect on overall survival were analyzed. Surgery-induced changes in the peritumoral network connectedness were evaluated in 7 patients with follow-up data.

**Results:**

Mean degree centrality was significantly higher in the lesional compared to the contralesional network (*P* = .032), indicating a tumor-induced effect on its local environment and reflecting high peritumoral network connectedness. Glioblastomas with a neural high epigenetic signature exhibited increased peritumoral network connectedness (*P* = .010), which was associated with decreased survival (*P* = .036). Postoperative peritumoral network connectedness tended to decrease, suggesting that surgical resection disrupts the functional communication between the tumor and its peritumoral environment.

**Conclusions:**

The role of network features in predicting patient survival suggests their clinical relevance as imaging biomarkers for assessing personalized treatment strategies, which may include targeting crucial nodes for disconnection or even neuromodulation of neural circuits.

**Key Points**

1. GBMs participate in a functionally connected network with their environment.
2. Higher peritumoral network connectedness is associated with decreased survival.
3. Surgery disrupts the functional communication within peritumoral networks.

**Importance of the Study**

Our study provides new insights into the integration of glioblastoma within the functional network of the peritumoral environment. Using a combination of advanced neuroimaging techniques and a network theory analysis, our findings show that glioblastoma not only forms functionally connected networks within the peritumoral region but also significantly alters the properties of these networks. High peritumoral network connectedness was associated with a distinct epigenetic signature and decreased overall survival. We identified that tumor connectedness is primarily driven by local, high-degree nodes and surgical resection appeared to disrupt this peritumoral network connectedness. Thus, our study presents a characteristic network property as an imaging biomarker that appears prognostically relevant emphasizing the need to consider connectivity in surgical planning for leveraging personalized treatment strategies.

Glioblastoma (GBM) is the most common malignant brain tumor of the central nervous system, with a prevalence of 47.60 per 100,000 and a worldwide annual incidence of 6 cases per 100,000, representing a 17.3% increase from 1990 to 2016.^1,2^ Despite advances in diagnostic and therapeutic strategies, the prognosis for patients with GBM remains dismal, with a mean overall survival (OS) of 15 months, even after maximal treatment, which typically includes surgical resection followed by adjuvant therapy.^3,4^

Recent discoveries have revealed that GBMs form functional connections with neurons in the surrounding environment, profoundly impacting both tumor behavior and neural circuitry.^5–8^ This neuron–tumor interaction not only drives tumor growth but also contributes to treatment resistance and recurrence.^9–11^ Glioma cells, especially at the tumor margins, often adopt neuronal-like transcriptional signatures, integrating into the peritumoral environment and hijacking neural progenitor programs.^6^

Recent work has further elucidated the role of neural integration in GBM, revealing distinct epigenetic signatures that divide tumors into low-neural and high-neural subtypes.^12^ Low-neural tumors were characterized by an inflammatory transcriptional signature, while high-neural GBMs exhibited upregulation of genes involved in synaptic integration and enrichment for transcriptomic programs characteristic of oligodendrocyte precursor cells and neural precursor-like cells. Moreover, high-neural GBMs demonstrated increased functional connectivity (FC) with their peritumoral environment, suggesting that the intricate interplay between tumor cells and the neural environment plays a pivotal role in tumor behavior. This interplay presents a unique opportunity to explore the disease’s underlying mechanisms, offering potential novel biomarkers and therapeutic targets.

Advanced neuroimaging techniques, such as resting-state fMRI, have become invaluable tools for investigating FC alterations within the peritumoral environment, providing insights into how the tumor is connected to its surrounding neural tissue.^13–15^ FC, which represents the synchronous neural activity between spatially separated brain regions, has emerged as a critical metric for characterizing the dynamic neural changes associated with GBM. Recent studies have shown that GBM influences peritumoral areas, creating regions of high and low FC, as evidenced by intracranial brain recordings during awake surgeries.^9^ The finding that local FC can be measured by subdural ECoG implies the possibility that using resting-state fMRI with adopting an agnostic approach can help investigate localized, tumor-related connectivity changes, aligning with known biological alterations induced by glioblastomas in their neuronal environment.

To analyze these FC changes within the peritumoral environment, network theory can be applied, offering crucial insights into the connectedness of the peritumoral network.^16,17^ This approach allows an estimation of the tumor’s impact on its immediate surroundings by viewing neural regions of interest (ROIs) as nodes within a graph, connected by edges that together build a complex network architecture. This network architecture holds key information about the network’s efficiency, controllability, and robustness. Clinically, applying network theory to functional networks enables the identification of critical nodes within the graph, providing insights into the dynamics of disease progression. Among the various network properties, degree centrality is of paramount importance, as it reflects each node’s connectedness and centrality within the network, indicating the importance for and the level of overall functional network performance.^18,19^

In this study, we sought to better understand the connectedness and network integration of GBM within the peritumoral environment by investigating local FC alterations and functional network dynamics in tumor-surrounding peritumoral areas. We show that GBM is functionally connected beyond the visible tumor margin, building a functionally connected network that appears to underlie a characteristic epigenetic signature. Our results suggest that higher peritumoral network connectedness is associated with poorer survival and that surgical resection can disrupt the peritumoral network by disconnecting its components.

## Materials and Methods

### Patients

**Figure 1.**
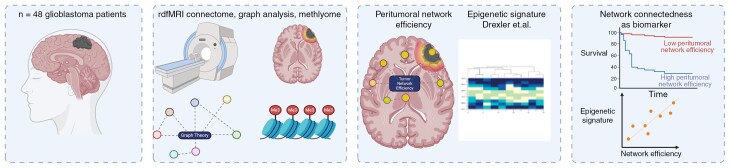
The experimental workflow integrating resting-state connectome and epigenetic signature. Peritumoral network connectedness was calculated from the functional connectome data using a graph theoretical approach, correlated to the neural epigenetic score (Drexler et al.) and used for survival analysis.

We retrospectively included 48 consecutive, histopathologically confirmed treatment-naîve, Isocitrate dehydrogenase-negative GBM patients (mean age: 65 ± 9 years) who underwent preoperative resting-state fMRI investigation in this study (Figure 1). Histopathological diagnoses were determined according to the WHO 2021 tumor classification. Of these 48 patients, 7 had received a postoperative fMRI assessment and were included for follow-up analyses (duration between initial and follow-up: mean = 18 ± 6 months). A methylation profiling of all GBMs was performed, deconvoluting the tumor DNA to evaluate GBMs’ neural signature. The study was approved by the local ethics committee of the Medical Faculty of the University of the RWTH Aachen (EK22-33, EK142-20), which requires no specific patients’ informed consent when retrospective patients’ data are analyzed, and conducted in accordance with the standards of Good Clinical Practice and the Declaration of Helsinki.

### MRI Data Acquisition

MRI examination was applied using a 3T Siemens Prisma MRI scanner equipped with a standard 20-channel head coil. The scanning protocol comprised a 3D T1 magnetization-prepared rapid acquisition gradient echo (MPRAGE) sequence was acquired: repetition time (TR) = 2300 ms, echo time (TE) = 2.32 ms, 192 slices with a slice thickness of 0.9 mm, flip angle = 8°, field of view (FoV) = 256*256 mm, voxel size = 0.957 × 0.957 × 0.9 mm^3^. For tumor identification purposes, a contrast-enhanced, 3D T1-weighted MPRAGE was acquired, with acquisition parameters being the same as for the non-contrast-enhanced MRPRAGE sequence. In addition, a 3D fluid attenuation inversion recovery (FLAIR) sequence was applied (TR = 4,800 ms, TE = 305 ms, number of slices = 160 with 1 mm slice thickness, FoV = 204*256 mm, voxel size = 0.9766 × 0.9766 × 1 mm), and RS-fMRI was implemented using echo planar imaging (EPI), including 300 whole-brain functional volumes, TR = 2200 ms, TE = 30 ms, number of slices = 36 with 3.1 mm slice thickness, flip angle = 90°, and FoV = 64*64 mm, voxel size = 3.125 × 3.125 × 3.565 mm^3^.

### Image Processing

Functional data were preprocessed using SPM12^20^ as implemented in Matlab 9.5^21^ according to an imaging protocol that was similarly applied and described in previous publications.^22,23^ Briefly, functional images were realigned to the mean functional volume, unwarped, and coregistrated to the structural image. Structural and functional images were segmented, bias-corrected and spatially normalized (multispectral classification), and functional images were smoothed with a 5 mm FWHM Gaussian kernel. Functional images were then slice-time corrected, movement-related time series were regressed out with ICA-AROMA,^24^ and data were high-pass filtered (>0.01 Hz). Image processing of postoperative data was performed in equivalence to this processing procedure.

### FC Analysis

**Figure 2.**
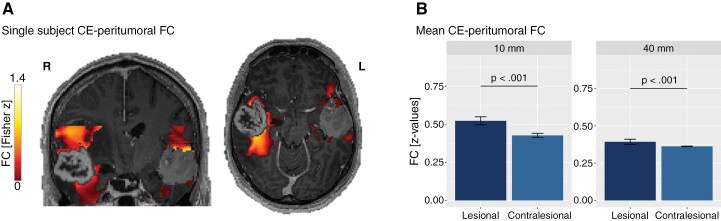
Lesional and contralesional tumor-FC in 10 mm and 40 mm peritumoral distance. Functional connectivity (FC) between contrast-enhancing (CE) tumor and the 10 mm and 40 mm peritumoral surrounding is visualized in a single subject with left-hemispheric GBM (A). Lesional as well as contralesional FC (between the mirrored tumor and mirrored peritumoral surrounding) is illustrated. Significant differences in group CE-peritumoral FC between the lesional and contralesional hemisphere are shown (B).

To evaluate changes in FC patterns between GBM and its peritumoral area, contrast-enhancing tumor lesions were segmented semi-automatically using the ITK-SNAP software version 3.4.0.^25^ Tumor volume in cm^3^ was computed and lesion masks were used as ROI to perform a seed-based correlation analysis: The mean timeseries of the tumor ROI was correlated to every voxel’s timeseries, creating a voxel-wise whole-brain correlation map to compute tumor to whole-brain FC. Correlations were Fisher *z* transformed and thresholded at *z* ≥ 0.02.^26^ Then, a 10 mm and 40 mm distance mask was created by dilating the tumor mask by 10 mm and 40 mm and subtracting the tumor area. When investigating FC between the tumor and the 40 mm distance mask, the 40 mm distance mask represented the region between tumor-border + 30 mm-dilation and tumor-border + 40 mm-dilation, reflecting a mask beginning at 30 mm distance from the tumor which is about 10 mm in size. The mean FC between the tumor and regions in 10 mm and 40 mm distance was computed, aiming to capture distance-dependent differences in tumor FC (Figure 2).

### Network Analysis

To better understand the functional relationship between the tumor and its peritumoral neighborhood, the tumor’s +10 mm and +40 mm surroundings were further analyzed by applying network theory. Specifically, the 10 mm and 40 mm peritumoral surrounding represented the whole 10 mm and 40 mm dilated area surrounding the tumor. This way, (size-depending) changes in network dynamics could be analyzed. Within the 10 mm and 40 mm surrounding, ROIs with 6 mm diameter were created, with the tumor itself being one ROI as well. Since tumor size varied between patients, created ROIs included enough voxels (each voxel’s size = 1.5 mm^3^) without covering too large an area to capture slight signal differences that might be present in one but no longer in the adjacent voxel. This way, we attempted to define the tumor margin based on tumor-like functional signals. The step distance between adjacent ROIs was 1 mm (approximately 0.67 voxels), and ROIs were directly adjacent, without overlapping voxels in different ROIs. ROIs were used as nodes within a 10 mm and 40 mm undirected network based on an edge weight of *z* ≥ 0.2 between nodes, with edge weights reflecting the Fisher *z*-transformed correlation coefficients between nodes (ROI pairs). Centrality measures represent quantitative metrics that measure the average importance of nodes in a network,^27^ indicating how well they can exchange information or resources. The degree of centrality captures a node’s direct influence on the local network based on the number of direct connections a node has. High levels of connectedness (high-degree centrality) enable nodes to communicate more efficiently by having more impact on the nodes they are connected with,^28^ resulting in better overall network performance. Conversely, low levels of connectedness (low degree centrality) can indicate less effective connections that can affect the functionality of the network. In this study, degree centrality computed node centrality specified by type for each node in the graph, counting the number of nodal connections each node has. The mean of degree centrality (MDC) was computed for the 10 mm and 40 mm network in each patient.

To include a “healthy” control (in the following referred to as contralesional) for peritumoral FC and network parameters (in the following referred to as lesional), tumor masks, 10 mm and 40 mm distance masks as well as 10 mm and 40 mm undirected networks were flipped towards the contralesional hemisphere. After computing a seed-based whole-brain FC map using the mirrored, “contralesional” tumor as seed, contralesional FC, and MDC were computed and compared to those of the lesional hemisphere. Then, the difference between lesional and contralesional MDC was computed as a measure of tumor-specific, peritumoral network connectedness, with positive values indicating a higher connectedness within networks of the lesional compared to the contralesional hemisphere.

Proof of principle: Supramarginal 10 mm, 40 mm, and FLAIR FC, MDC, and peritumoral connectedness.

Due to emerging alternative concepts regarding the surgical treatment of GBM extending “maximum safe resection of the contrast enhancing tumor” to different definitions of supramarginal resection, e.g. 10 mm beyond contrast-enhancement or FLAIR-based, potential benefits have to be weighed against the associated neurological risks in order to ethically justify this approach. To visualize similarities and differences between these concepts, contrast non-enhanced FLAIR-based hyperintensities were segmented, creating a binary FLAIR peritumoral mask. Equivalent to 10 mm and 40 mm analyses, FLAIR peritumoral masks were mirrored to the contralesional hemisphere, and FLAIR FC, MDC, and peritumoral network connectedness were computed.

### DNA Methylation Processing and Deconvolution

DNA was extracted from tumors, extracellular vesicles, and bulk plasma, and analyzed for genome-wide DNA methylation patterns using the Illumina EPIC (850k) array. The processing of DNA methylation data was performed with custom approaches (10.1038/nature26000 and 10.1007/s00401-018-1879-y). All IDAT files were processed using the preprocess Illumina (minfi, v.1.40.0). Probes with detection *P* values < .01 were kept for further analysis. Probes with < 3 beads in at least 5% of samples, all non-CpG probes, SNP-related probes, and probes located on X and Y chromosomes were discarded. For determining the epigenetic neural signature (10.1038/s41591-024-02969-w), we used the cell-type-specific methylation signature available from Moss et al.^29^ consisting of 25 cell-type components. We used the original implementation of Moss et al. to perform cell-type deconvolution using non-negative least square linear regression.

### Statistics

All statistical analyses were performed with SPSS 27.^30^ Normal distributions were explored for the whole sample (*N* = 48), and extreme outliers with values deviating 3 or more standard deviations from the mean were corrected by replacing these values by mean ± 3 standard deviations on the respective variable.

#### FC Analysis:

Local FC differences between the tumor and regions in 10 mm and 40 mm distance as well as FC differences between lesional and contralesional hemispheres were analyzed by applying a Repeated Measures Analysis of Covariance (RM ANCOVA), including FC as dependent variable, distance and hemisphere as within-subject factors, and tumor volume as covariate.

#### Network Analysis:

MDC, a measure of nodal connectedness, is believed to reflect network resilience and may indicate (maladaptive) hyperconnectivity in the peritumoral environment. MDC differences between lesional and contralesional networks were analyzed using a RM ANCOVA, including MDC of the 10 mm and 40 mm networks as dependent variables, hemisphere as within-subject factor, and tumor volume as covariate.

#### GBM Epigenetic Signature:

 To investigate the association between the tumor’s biological background and network properties within the peritumoral surroundings, a partial correlation analysis was applied, entering neural epigenetic score and peritumoral network connectedness as variables, while controlling for tumor volume.

#### Survival Analyses:

To examine the importance of network’s resilience for survival prediction in GBM, peritumoral network connectedness was analyzed regarding its predictive power for OS. Multivariate Cox regression analyses were applied, including days of survival as time and death as status variables. 10 mm and 40 mm peritumoral network connectedness were entered as independent predictor variables along with Methyl-Guanin-DNA-Methyl-Transferase (MGMT) status, adjuvant treatment, preoperative Karnofski performance score, and age.

#### Follow-up Analyses:

Single-case postoperative changes in peritumoral network connectedness were evaluated in 7 patients, performing descriptive statistics.

#### Proof of Principle:

Supramarginal 10 mm, 40 mm, and FLAIR FC, MDC, and peritumoral connectedness.

Hemispheric FC differences between lesional and contralesional tumor-to-FLAIR were analyzed in 33 patients with FLAIR masks within one hemisphere, applying a RM ANCOVA, including FLAIR FC as dependent variable, hemisphere as within-subject factors, and tumor volume as covariate. Lesional and contralesional MDC were compared using a RM ANCOVA, including MDC of the FLAIR network as dependent variable, hemisphere as within-subject factor, and tumor volume as covariate.

## Results

### GBM Induces Local FC Alterations in the Peritumoral Environment

First, the FC between a GBM and its peritumoral environment was evaluated by comparing FC at 10 mm and 40 mm distances from the tumor margin. To determine whether these FC patterns were tumor-specific, they were compared to the corresponding contralesional FC. Multivariate analyses revealed a distance-dependent difference between lesional and contralesional FC, suggesting that FC alterations within the tumor’s environment were indeed tumor-induced (*F*(1,46) = 8.352, *P* = .006). Specifically, post hoc contrasts showed that lesional FC between the tumor and areas in 10 mm distance was significantly increased compared to the corresponding contralesional FC (*F*(1,46) = 11.124, *P* = .002, mean_10mm_ = 0.52, mean_40mm_ = 0.43). This effect was not found at the 40 mm distance (*F*(1,46) = 2.756, *P* = 0.104, mean_10mm_ = 0.39, mean_40mm_ = 0.36), indicating tumor-induced local FC alterations predominantly in tumor-adjacent areas (Figure 2).

**Figure 3.**
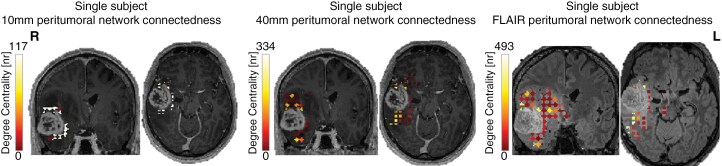
10 mm, 40 mm, and FLAIR peritumoral network connectedness. This figure visualizes peritumoral network connectedness in one single subject, contrasting the 10 mm (left panel), 40 mm (middle panel), and FLAIR-based (right panel) differences between lesional and contralesional degree centrality. Central, highly connected peritumoral nodes are indicated by a high degree centrality value and less connected nodes by a low degree centrality value.

Based on these findings, we investigated whether tumor-specific FC changes can be captured within the glioblastoma FLAIR area. Similarly, FLAIR-based analyses revealed increased FLAIR FC within the lesional as compared to contralesional hemisphere (*F*(1,31) = 5.735, *P* = .023). Likewise, FLAIR-based lesional MDC was higher than contralesional MDC (*F*(1,31) = 8.946, *P* = .005) (Figure 3).

### GBM Peritumoral Network Connectedness Reveals a Distinct Epigenetic Signature

**Figure 4.**
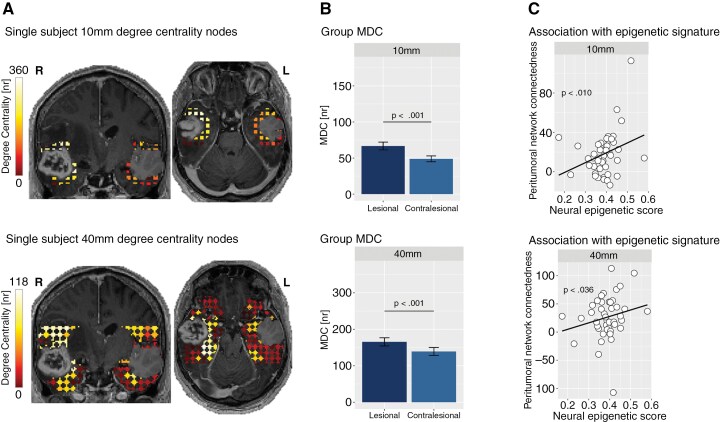
Lesional and contralesional degree centrality in 10 mm and 40 mm networks. The figure shows degree centrality within the lesional and contralesional hemisphere for both the 10 mm (upper panel) and 40 mm (lower panel) networks. Degree centrality nodes are visualized in a single subject (A), indicating central, highly connected peritumoral nodes with high degree centrality values and less connected nodes with low degree centrality values. Significant differences in group mean degree centrality (MDC) between the lesional and contralesional hemisphere are shown (B). (C) The partial correlation between neural neuronal GBM signature and peritumoral network connectedness, as indicated by the difference between lesional and contralesional MDC, is illustrated..

After establishing that GBMs form functionally connected networks with their peritumoral regions, we aimed to understand how the tumor may alter the properties of these networks. To investigate this, we analyzed MDC in the 10 mm and 40 mm networks and compared it between the lesional and contralesional hemispheres. Multivariate analyses revealed that MDC was significantly higher in the lesional compared to the contralesional hemisphere, suggesting a tumor-induced effect on networks within its peritumoral environment (*F*(2,45) = 3.709, *P* = .032). This effect was evident in both the 10 mm and 40 mm network (10 mm: *F*(1,46) = 7.381, *P* = .009, mean_lesional_ = 66.63, mean_contralesional_ = 48.77; 40 mm: *F*(1,46) = 4.794, *P* = .034, mean_lesional_ = 165.55, mean_contralesional_ = 139.19; Figure 4).

The difference between lesional and contralesional MDC was computed as a measure of tumor-specific, peritumoral network connectedness, with positive values indicating a higher connectedness within the lesional compared to the contralesional network. Given previous findings that GBMs exhibit distinct neural epigenetic signatures, the association between peritumoral network connectedness and its neural epigenetic signature was analyzed. Partial correlation analyses revealed a significant positive correlation, indicating that tumor-adjacent areas with higher peritumoral network connectedness also possess a higher neural epigenetic score (10 mm: *r* = 0.389, *P* = .010; 40 mm: *r* = 0.320, *P* = .036; Bonferroni-corrected significance for multiple comparisons *P*_adj_ = .025; Figure 4).

### Higher Peritumoral Network Connectedness Decreases Survival and can be Effectively Disrupted by Surgical Resection

**Figure 5.**
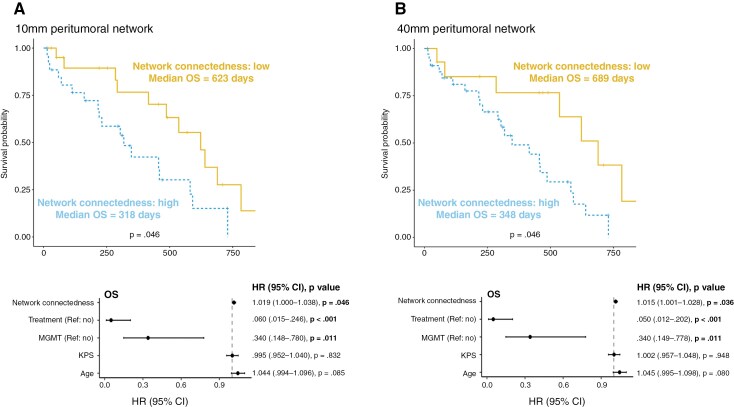
Kaplan–Meier curves and Forest plots for Cox proportional hazards models. This figure visualizes the effect of 10 mm (A) and 40 mm (B) peritumoral network connectedness on overall survival (OS). In the upper row, Kaplan–Meier curves of dichotomized peritumoral network connectedness show the difference in survival days depending on the strength of peritumoral network connectedness. In the lower row, forest plots are illustrated, reporting the hazard ratio and the 95% confidence intervals of the hazard ratio for each covariate in the Cox proportional hazards model for overall survival (OS) included 10 mm and 40 mm peritumoral network connectedness, adjuvant treatment, methyl-guanin-DNA-methyl-transferase (MGMT) methylation, preoperative Karnofsky performance score (KPS), and age.

The integration of GBM into neural circuits is crucial for the tumor’s resistance to treatment and impacts OS. To assess the relevance of peritumoral network connectedness for OS, tumor-near 10 mm and tumor-far 40 mm network connectedness were analyzed using a Cox regression model, accounting for MGMT status, adjuvant treatment, age, and preoperative Karnofsky scores. Results revealed that peritumoral network connectedness within the 10 mm network was a significant predictor for OS, along with MGMT status and adjuvant treatment (χ^2^(5) = 35.854, *P* < .001). Specifically, the regression model indicated a higher peritumoral network connectedness to be associated with shorter OS (*B* = 0.014, *P* = .036), as were GBMs without MGMT methylation (*B* = −1.078, *P* = .011) and patients without adjuvant treatment (*B* = −2.810, *P* < .001). Similarly, results revealed that peritumoral network connectedness within the 40 mm network was a significant predictor of OS and associated with shorter OS, along with MGMT methylation and adjuvant treatment (χ^2^(5) = 36.561, *P* < .001, peritumoral network connectedness: *B* = 0.019, *P* = .046; MGMT methylation: *B* = −1.078, *P* = .011; adjuvant treatment: *B* = −3.000, *P* < .001, Figure 5).

**Figure 6.**
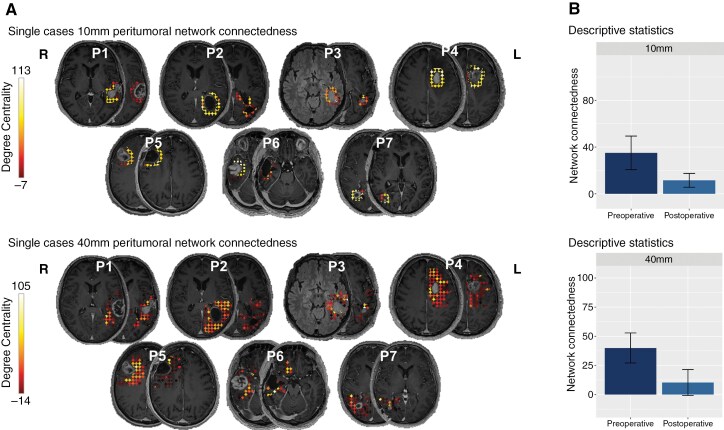
Postoperative changes in 10 mm and 40 mm peritumoral network connectedness. Pre- and postoperative peritumoral network connectedness is visualized for 7 patients with follow-up assessment (A), regarding both the 10 mm (upper panel) and 40 mm (lower panel) networks, indicating central, highly connected peritumoral nodes with high degree centrality values and less connected nodes with low degree centrality values. (B) Group mean differences and standard error between the preoperative and postoperative peritumoral network connectedness are illustrated.

In a subset of 7 patients with available postoperative resting-state fMRI exams, the impact of surgical resection on peritumoral network connectedness was investigated by comparing 10 mm and 40 mm peritumoral network connectedness before and after surgery. Surgical resection resulted in a decrease in peritumoral network connectedness in the 10 mm network (preoperative: mean = 31 ± 28; postoperative: mean = 12 ± 16) and in the 40 mm network (preoperative: mean = 40 ± 34; postoperative: mean = 10 ± 29). These findings suggest that surgical intervention may effectively disrupt communication within the peritumoral network, potentially contributing to the functional disconnection of GBM from its surrounding brain tissue (Figure 6).

## Discussion

Our study offers novel insights into the integration of GBM within the functional network of the peritumoral environment. Utilizing advanced neuroimaging and network theory analysis, we demonstrate that GBM not only forms functionally connected networks with the surrounding brain tissue but also significantly alters the properties of these networks. For this purpose, we adopted an agnostic approach to investigate tumor-related FC changes within peritumoral 10 mm, 40 mm, and FLAIR areas, aiming to capture the biological background of glioblastoma’s synaptic integration into neuronal circuits. These tumor-induced FC alterations include increased peritumoral network connectedness, which is linked to distinct epigenetic signatures and reduced OS. Surgical resection could effectively reduce this peritumoral network connectedness, disrupting the tumor-to-peritumoral connectivity.

### Tumor-Induced FC and Peritumoral Network Connectedness

Our findings underscore the profound influence of GBM on adjacent brain regions, extending far beyond the visible tumor margins. The heightened FC between the tumor and its immediate 10 mm peritumoral region, in contrast to the less pronounced FC in the 40 mm region, illustrates a distance-dependent integration of the tumor into the neural network. This finding is consistent with emerging evidence that GBM cells at the tumor borders adopt neuronal-like properties, enabling them to integrate into and potentially co-opt neural circuits in the peritumoral environment.^6^ The elevated MDC observed in the lesional hemisphere further supports the hypothesis that GBM drives maladaptive hyperconnectivity, contributing to its invasive behavior and resistance to conventional therapies.^19,31^

### Epigenetic Signature of Increased Peritumoral Network Connectedness

Our results further revealed that increased peritumoral network connectedness is associated with distinct epigenetic signatures. Specifically, regions with high peritumoral network connectedness are enriched with cortical neurons, suggesting that GBM not only disrupts the FC of surrounding brain tissue but also modulates its cellular composition and epigenetic landscape. This indicates that the integration of GBM into neural circuits is not merely a passive consequence of tumor growth but is actively driven by epigenetic reprogramming, enhancing the tumor’s ability to interact with and manipulate the neural environment. The association between higher neural epigenetic signatures and increased peritumoral network connectedness may reflect a shift in the local environment, fostering conditions that are more conducive to tumor growth and resistance to therapy. These findings are in line with a recent work by Drexler et al. showing that high-neural GBMs show worse OS.^12^

### Impact on Survival—Resect to Disconnect

We further explored the role of peritumoral network connectedness on survival, demonstrating that higher peritumoral network connectedness correlates with shorter OS, highlighting the clinical significance of GBM’s integration into neural circuits. The association between increased peritumoral network connectedness and decreased survival suggests that the robustness of these tumor-associated networks may contribute to the tumor’s resistance against conventional therapies and its ability to recur after treatment. These findings are sound, since increased MDC signifies a shift in the overall connectedness of a network, also meaning more redundant pathways, making the network more resilient to random failures. These findings are in line with previous studies showing that GBM patients with high functionally connected voxels derived from MEG recordings show worse survival,^6^ or that central nodes are critical for altering proliferation rates in temozolomide-resistant glioma cell lines and for predicting survival in glioma patients.^32^

Surgical resection is one of the most effective strategies for disrupting these resilient networks. Our postoperative analysis revealed a reduction in peritumoral network connectedness following surgical resection, both in the 10 mm and 40 mm peritumoral regions. These findings support the concept that surgery plays a crucial role in disrupting the FC between GBM and its adjacent brain tissue, potentially disconnecting the tumor’s integration into neural circuits and improving patient outcomes. These findings are in line with recent research, demonstrating that effective altering of networks’ robustness can be achieved based on removing nodes with high degree and high betweenness,^32^ and that patients with higher neuronal score may benefit more from complete resection,^12^ following the new coined paradigm of *resect to disconnect.*

In clinical practice, these results emphasize the potential value of utilizing FC measures and network theory analysis approaches to visualize GBM network integration. We showed that glioblastoma-specific network connectedness was detectable within the FLAIR area as well, possibly creating a putative target that can guide the extent of resection in the recently reviving era of supramarginal resections. By identifying network nodes crucial for network robustness and efficiency, neurosurgeons can strategically target tumor resection to disconnect GBM’s connectivity with the peritumoral environment, thereby optimizing therapeutic strategies and personalizing surgical treatment (Supplementary Figure S1). Future work should include a broad analysis of GBM networks to topological attacks to better detect relevant targets for therapeutic solutions.

### Limitations of the Study

One limitation of this study is the limited number of patients, and the lack of case-by-case postoperative imaging for every patient creates an important limitation that should be taken into consideration. Although resting-state fMRI is a well-established imaging modality, the robustness of results could be increased by performing multiple investigations. Finally, the retrospective nature of the study created a possible selection bias that should be accounted for.

## Conclusion

Our findings provide a comprehensive understanding of the relationship between GBM, FC, and network properties. The role of network features in predicting patient survival underscores their clinical relevance as imaging biomarkers for assessing personalized treatment strategies. These strategies may include targeted disconnection of crucial nodes to achieve modulation of cancer-neural circuits.

## Supplementary Material

noaf101_Supplementary_Figure_S1

noaf101_Supplementary_Figure_Legend

## Data Availability

De-identified, original MR images and source code of the applied pre- and post-processing protocol will be stored in the Coscine repository (https://www.coscine.de) and will be made available upon reasonable request.

## Notes

*Conflict of interest statement.* J.O.: Institutional research funding from European Association of Neurosurgical Societies, Stiftung Universitätsmedizin; M.W.: Reimbursement for lectures or travel support from Bracco Imaging, Medtronic, Siemens Healthcare, Stryker Neurovascular. Consulting fees from Stryker Neurovascular. Institutional research funding or educational exhibits from Ab medica, Acandis, Bayer, Bracco Imaging, Cerenovus, Codman Neurovascular, Dahlhausen, Kaneka Pharmaceuticals, Medtronic, Mentice AB, Microvention, Phenox, Siemens Healthcare; K.J.: None declared; J.M.K.: None declared; A.M.-B.: None declared; U.M.-B.: None declared; H.A.H.: None declared; H.C.: None declared; J.B.: None declared; A.B.: None declared; F.R.: None declared; R.D.: None declared; D.H.H.: None declared; D.D.: None declared.
