# Supplementary figures and images for: High peritumoral network connectedness in glioblastoma reveals a distinct epigenetic signature and is associated with decreased overall survival
Source: Neuro Oncol. 2025 Apr 15;27(10):2564–73. doi: 10.1093/neuonc/noaf101 (PMC12833544; doi:10.1093/neuonc/noaf101)

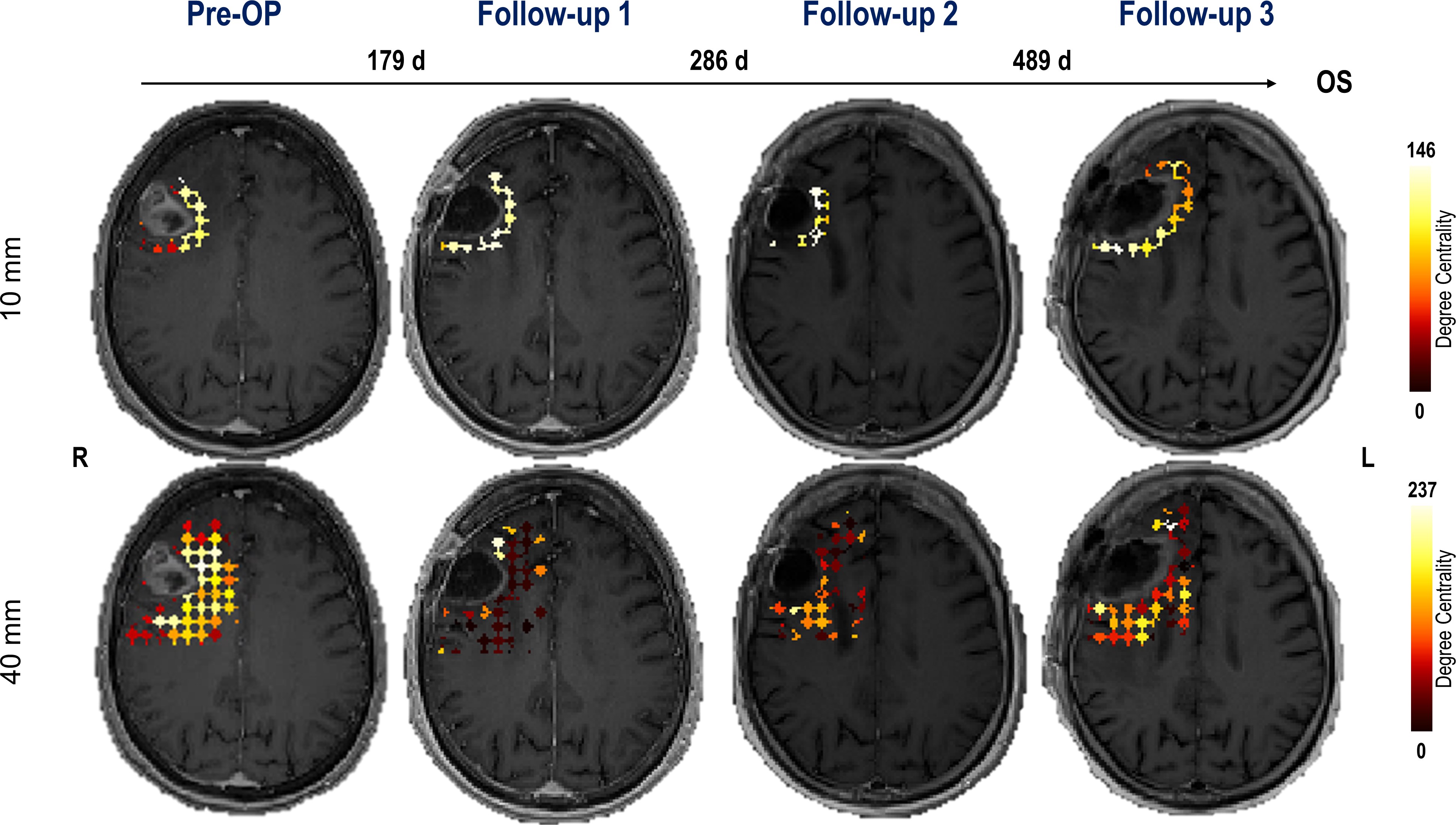

Supplement: noaf101_Supplementary_Figure_S1 [file noaf101_supplementary_figure_s1.jpeg]
