# Supplementary material for: High peritumoral network connectedness in glioblastoma reveals a distinct epigenetic signature and is associated with decreased overall survival
Source: Neuro Oncol. 2025 Apr 15;27(10):2564–73. doi: 10.1093/neuonc/noaf101 (PMC12833544; doi:10.1093/neuonc/noaf101)
Supplement: noaf101_Supplementary_Figure_Legend [file noaf101_supplementary_figure_legend.docx]

***Supplementary Figure S1.*** *Single case longitudinal changes in peritumoral network connectedness. In the case of this patient with long-term follow-up of 489 days (d), peritumoral network connectedness (the difference between lesional and contralesional degree centrality) of the 10mm and 40mm networks are mapped across preoperative (Pre-OP) and follow-up assessments. Within the 40mm network, peritumoral network connectedness appeared to initially decrease from pre- to postoperatively, with the exception of a few nodes that were still highly connected. Starting from these nodes, the connection strength of the surrounding nodes seemed to gradually increase again in later follow-up assessments, possibly indicating a stabilization of tumor-specific activation level. Within the immediate 10mm surrounding, such an increase in peritumoral network connectedness appeared to be detectable already earlier (Follow-up 1), while it was only measurable later (Follow-up 2) in the wider, more remote 40mm network*.
